# Diffraction structural biology – a new horizon

**DOI:** 10.1107/S0909049513025478

**Published:** 2013-10-11

**Authors:** Takashi Yamane, John R. Helliwell, John E. Johnson, Noritake Yasuoka, Noriyoshi Sakabe

**Affiliations:** a Nagoya Industrial Science Research Institute, 1-13 Yotsuya-dori, Chikusa-ku, Nagoya 464-0819, Japan; b School of Chemistry, University of Manchester, Manchester M13 9PL, UK; c Scripps Research Institute, San Diego, CA, USA; d AIST Kansai Center, 1-8-31 Midorigaoka, Ikeda, Osaka 563-8577, Japan; e Photon Factory, KEK, 1-1 Oho, Tsukuba, Ibaraki 305-0801, Japan

## Abstract

An introductory overview to the special issue papers on diffraction structural biology in this issue of the journal.

This issue of the *Journal of Synchrotron Radiation* presents articles submitted in the context of the 4th International Symposium on Diffraction Structural Biology (ISDSB2013) held at the Fukiage Hall in Nagoya on 26–29 May 2013. It was the fourth in the series of ISDSB symposia initiated in 2003 by the Japan Society for the Promotion of Science (JSPS) and specifically by the University–Industry Cooperative Research Committee (#169) chaired by Professor Noriyoshi Sakabe. The previous conference was held in France, close to Paris at Paris-Sud University (Orsay) and the synchrotron radiation facility SOLEIL, chaired by the late Roger Fourme. ISDSB2013 came back to Japan again.

The basic concept of the ISDSB symposia is, firstly, to bring together researchers using diffraction and crystallography, and more generally interactions of X-rays, electrons and neutrons with matter, in the study of structural biology, and, secondly, within this domain, to facilitate the interaction between academic and industrial researchers. The interfaces with other active fields in biological ultra-structure using microscopies and spectroscopies (in particular nuclear magnetic resonance) are also respected as vital for the growth of a more systematic understanding of biological function based on structure. The scientific topics covered in ISDSB2013 included the following sessions: (1) synchrotron radiation and free-electron lasers; (2) new methodology and instrumentation; (3) and (4) drug design; (5) electron microscopy; (6) tomography and imaging; (7) neutron diffraction and hydration structure; (8) membrane proteins and macromolecular complexes; (9) protein structure and dynamics. A total of 207 participants, with a number of young scientists and PhD students, from 14 countries took part. The speakers included two Nobel Prize winners, Thomas Steitz and Brian Kobilka, three plenary lecturers (Yoshinori Fujiyoshi, Paul Langan and Ian Wilson) and 31 invited speakers. A poster session was also held and 80 posters were presented. Thirteen commercial and industrial companies presented exhibitions, and a sponsored lunch seminar was also given.

The symposium was supported jointly by the #169 Committee of JSPS and by many industrial companies as well as generous individuals. We wish to express our sincere thanks to the University–Industry Cooperation and Research Program Division of the JSPS, to the Industry Club of Japan for their financial contribution through JSPS to print this issue, to the Naito Foundation for supporting the travel fees, and to all companies and persons who contributed their donations and cooperation.

